# Spontaneous Hemorrhagic Pericardial Effusion as a Consequence of Apixaban Utilization for New-Onset Atrial Fibrillation

**DOI:** 10.7759/cureus.56510

**Published:** 2024-03-19

**Authors:** Dhaval Trivedi, Adrian Michael Lorenzana, Krystyna Bronchard, Bharath Reddy

**Affiliations:** 1 Internal Medicine, New York Presbyterian - Brooklyn Methodist Hospital, Brooklyn, USA; 2 Cardiology, New York Presbyterian - Brooklyn Methodist Hospital, Brooklyn, USA; 3 Cardiac Electrophysiology, New York Presbyterian - Brooklyn Methodist Hospital, Brooklyn, USA

**Keywords:** eliqius, apixaban, pericardiocentesis, pericardial effusion, cardiology

## Abstract

Although anticoagulation stands as a standardized therapeutic approach for mitigating thrombotic risks in atrial fibrillation, the potential for bleeding associated with direct oral anticoagulants (DOACs) is consistently weighed in the risk/benefit analysis prior to initiating therapy for non-valvular atrial fibrillation. While the typical bleeding risks from DOACs predominantly affect the gastrointestinal system, occurrences of spontaneous hemorrhagic pericardial effusions are rare. This case presentation illustrates a patient developing spontaneous hemorrhagic pericardial effusion four days after commencing apixaban therapy and subsequent management.

## Introduction

A pericardial effusion is a fluid accumulation within the pericardium that surpasses the expected physiological range. Effusions are typically detected as incidental findings during echocardiography or following diagnostic studies on a symptomatic patient. Typically, if the patient is asymptomatic, most pericardial effusions do not require specific treatment. Large pericardial effusions have an increased risk of developing into cardiac tamponade, which requires pericardiocentesis as the curative intervention [1].

Although pericardial effusions are common, few epidemiological data exist on their incidence and prevalence of effusions in the clinical setting. Most studies, when evaluating the developed versus developing countries, found that idiopathic etiology is the leading cause of pericardial effusion in the developed countries and tuberculosis in the developing countries. Analysis and further epidemiological studies are mainly dependent on the epidemiological background, the institutional setting, and the availability and access to subspecialties [2].

Pericardial effusions may be either an acute or chronic accumulation of fluid within the pericardial space. In the acute setting, due to the limited elasticity within the pericardium, only 100-150 milliliters are necessary to cause cardiac tamponade. In the chronic setting, the effusion may become one to two liters in size before causing cardiac tamponade as long as the accumulation is gradual and the pericardium has the facilities time to stretch and accommodate the volume [3]. 

Effusions may be broadly characterized into transudative, exudative, or sanguineous types; sanguineous-type effusions are associated with iatrogenic, neoplastic, ischemic, or idiopathic etiologies [4]. Conversely, drug-induced causes are rare. In particular, medications such as DOACs can worsen pre-existing hemopericardium by promoting further bleeding incited by the aforementioned etiologies but are not frequently attributed as a primary cause [5]. 

Direct oral anticoagulants, which are the mainstay treatment for non-valvular atrial fibrillation and venous thromboembolism, have been described to carry a high risk for gastrointestinal bleeding, but a lower risk for intracranial and life-threatening hemorrhages compared to warfarin. Spontaneous hemopericardium has been found to occur in 2.5-11% of patients receiving anticoagulation with DOACs [6]. In this case presentation, we highlight an aggressive and insidious onset of spontaneous hemopericardium upon initiating apixaban therapy.

## Case presentation

A 79-year-old male, who was a former smoker with a 50-pack-year history, with a medical history of asthmatic bronchitis, chronic kidney disease (stage 3B, eGFR 40 ml/min/1.73 m^2^), hypertension, hyperlipidemia, benign prostate hyperplasia, type 2 diabetes mellitus, varicose veins, and recently diagnosed atrial fibrillation presented to the emergency department with dyspnea on exertion with an associated productive cough with white sputum. He endorsed experiencing worsening exertional dyspnea with minimal ambulation and positional orthopnea, requiring two pillows. He denied leg pain or swelling and has had no recent flights. Notably, atrial fibrillation was recently diagnosed three days before admission and he was subsequently started on renally-dosed apixaban for anticoagulation.

**Figure 1:**
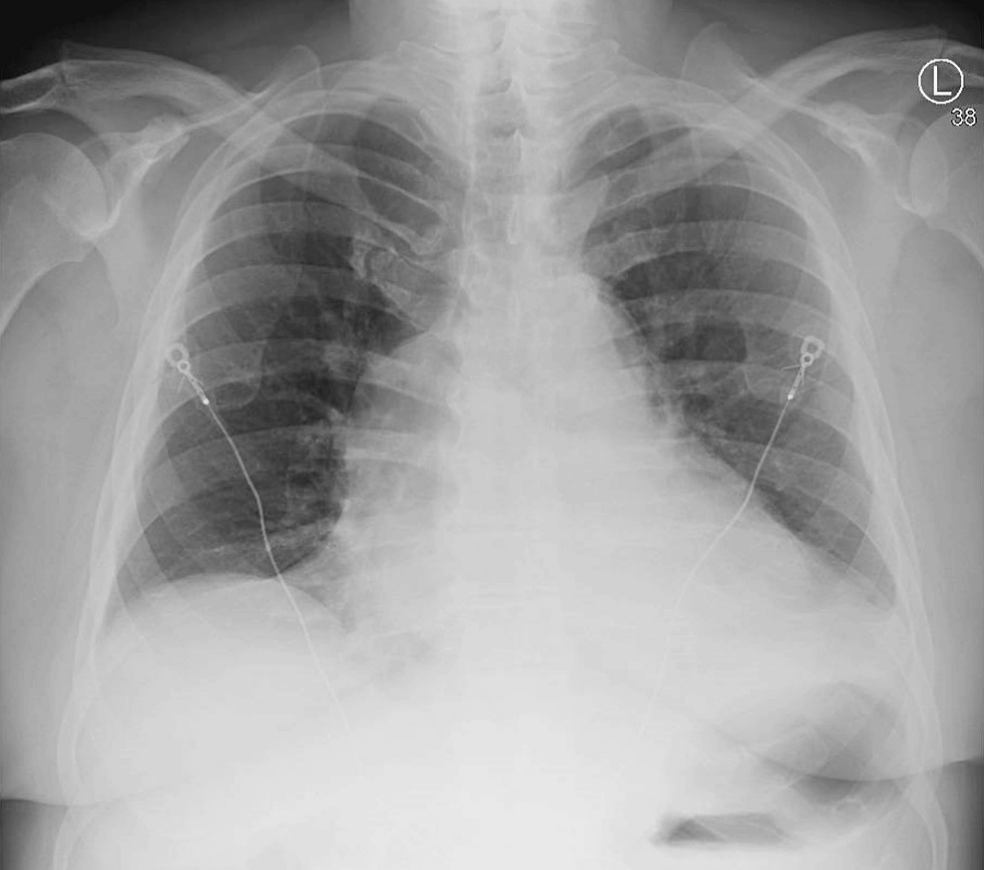
Chest X-ray (PA view) PA: Posterianterior

**Figure 2:**
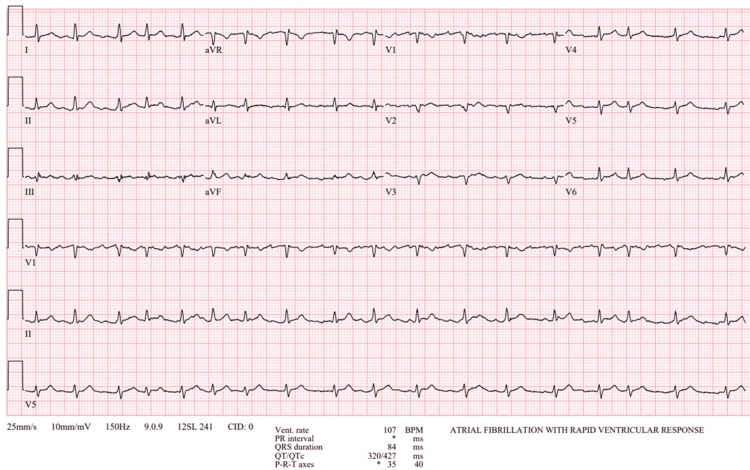
Initial electrocardiogram

In the emergency department, vital signs were as follows: temperature 36.3°C, heart rate 96 beats/minute, respiratory rate 18 breaths/minute, blood pressure 125/82, and SpO2 96% on room air. Physical examination was remarkable for an irregularly irregular rhythm. Chest X-ray showed a normal cardiomediastinal silhouette (Figure 1). The electrocardiogram showed atrial fibrillation with rapid ventricular response and low-voltage QRS (Figure 2).

**Figure 3:**
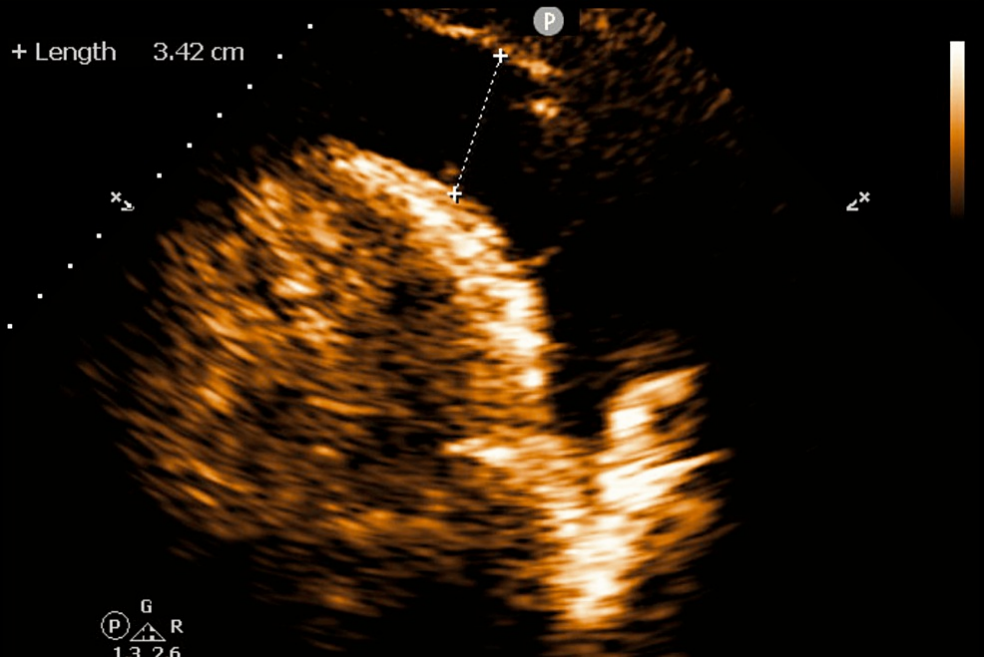
Transthoracic echocardiogram The echocardiogram in the Parasternal Long Axis View (PLAX) is showing a large pericardial effusion.

Given the clinical concern that the patient's dyspnea on exertion was due to heart failure versus community-acquired pneumonia, empiric antibiotic coverage with ceftriaxone and azithromycin was initiated. Subsequently, a transthoracic echocardiogram was performed for an evaluation of his systolic function, which revealed a left ventricular ejection fraction of 55-60% and a large pericardial effusion (Figure 3) with diastolic compression of the right atrium and ventricle (Video 1).

**Video 1:** Large pericardial effusion with right atrial and ventricular compromise (apical 4 chamber view)

With the concern of a hemodynamically significant pericardial effusion, the patient was immediately moved to the cardiac critical care unit. Upon arrival, the patient developed worsening hypotension and a physical exam revealed cold/clammy extremities, faint heart sounds, and jugular venous distension. Laboratory studies were notable for worsening renal failure, elevated liver function tests, leukocytosis, and severe lactic acidosis. Given the concern for cardiac tamponade, the patient was emergently taken to the catheterization lab for emergent pericardiocentesis.

While in the catheterization lab, the patient became unresponsive with asystole/pulseless electrical activity (PEA) arrest, and cardiopulmonary resuscitation was initiated. The patient was intubated and bag-mask ventilation was performed. Return of spontaneous circulation was achieved after 8.5 minutes with the following pharmacological interventions: 1 milligram of intravenous epinephrine, 50 mEq intravenous sodium bicarbonate, and 25 grams of intravenous dextrose. Pericardiocentesis was performed during Advanced Cardiac Life support (ACLS) and a pericardial drain yielded 500 cc bloody output.

**Figure 4:**
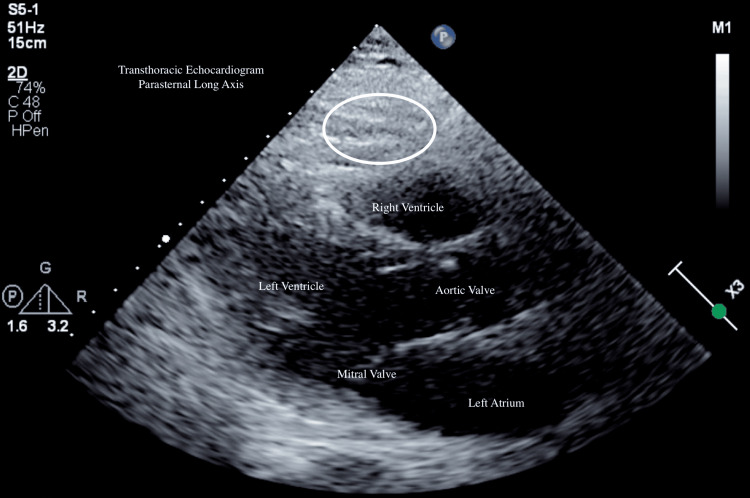
Interval transthoracic echocardiogram (PLAX) PLAX: Parasternal Long Axis View The white circle shows the resolved pericardial effusion.

**Figure 5:**
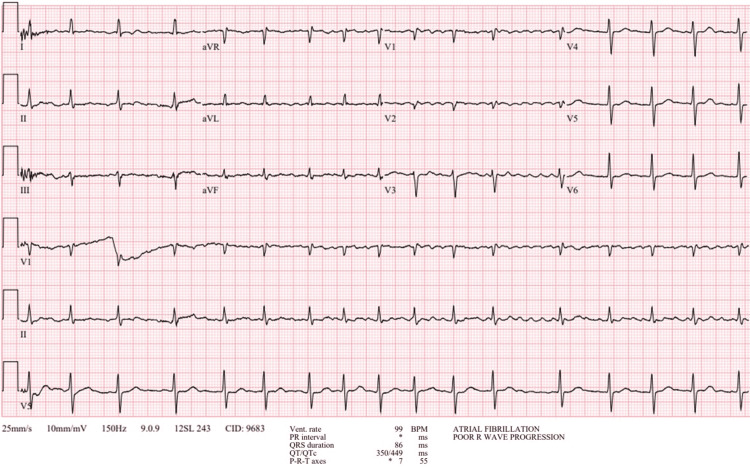
Post-pericardiocentesis EKG (atrial fibrillation without low voltage QRS)

Right heart catheterization was performed simultaneously with pericardiocentesis, allowing exclusion of constriction, which revealed normal pulmonary artery pressures, elevated pulmonary capillary wedge pressure, and normal cardiac output/index. The patient was successfully extubated the following day and slowly weaned off vasopressor support. A repeat echocardiogram was performed post-extubation which showed resolution of pericardial effusion (Figure 4, white circle), normal left ventricular systolic function, normal right ventricular size, and no diastolic compromise of the right atrium (Video 2). A repeat electrocardiogram revealed atrial fibrillation without low-voltage QRS (Figure 5).

**Video 2:** Resolved large pericardial effusion (apical 4 chamber view)

Pericardiocentesis fluid analysis revealed no fungi, acid-fast bacilli, or organisms; cytology revealed predominantly blood without malignant cells. The patient's clinical course was further complicated by acute renal failure requiring continuous veno-venous hemodialysis (CVVHD) through a right internal jugular non-tunneled dialysis catheter. Course with further complicated by the development of dyspnea, hypertension, and tachycardia requiring esmolol infusion. Esmolol improved his persistent hypertension and tachycardia and he was titrated to oral carvedilol. After four days, CVVHD was stopped as the creatinine stabilized and the patient clinically improved and voided clear urine. Intravenous furosemide was used to further aid in diuresis and daily metabolic panels were evaluated for renal function. Creatinine progressively continued to rise and furosemide was stopped. Eventually, a tunneled hemodialysis catheter was placed for short-term dialysis treatment.

Upon discharge, the electrocardiogram continued to show atrial fibrillation and renally-dosed apixaban was restarted for anticoagulation with amiodarone for rhythm control. Discharge follow-up was established with electrophysiology for radiofrequency ablation of atrial fibrillation. In regards to the patient's renal function, he became non-oliguric with slow recovery in renal function and his dialysis was tapered to once a week and planned for tunneled catheter removal. In the case of recurrent bleeding, a patient-centered approach for a left atrial appendage occlusion device was discussed.

## Discussion

Although bleeding is the most common complication in the initiation of DOACs, hemorrhagic pericardial effusions remain a rare entity in this subset [7]. In a systematic review, hemorrhagic pericardial effusion was found to be a rare complication of DOAC usage. Out of the identified 26 cases, five were associated with apixaban, nine with dabigatran, and 12 with rivaroxaban; two cases were associated with mortality, and 24 required pericardiocentesis [8]. Given the high risk of mortality associated with untreated hemorrhagic pericardial effusion, extensive care must be taken to ensure early diagnosis and treatment of new-onset signs and symptoms of pericardial effusion post-apixaban utilization. 

The etiology of hemorrhagic pericardial effusion evolves from the pharmacodynamics of apixaban. Apixaban is a highly selective direct factor Xa inhibitor, blocking the propagation phase of the coagulation cascade, which involves the production of large amounts of thrombin to promote clot formation. Trauma within the vascular system activates platelets through exposed endothelium or collagen and activates that intrinsic pathway of the coagulation cascade, which involves Factors XII, XI, IX, VIII and finally activating Factor X to Xa. Notably, with apixaban pharmacokinetics, absorption shows 50% bioavailability, which is mainly metabolized by (CYP)3A4 with elimination occurring in both urinary and gastrointestinal systems [9]. In atrial fibrillation, clot formation is mediated by increased turbulence and left atrial stasis, especially in the left atrial appendage (LAA), incurred by irregular left atrial conduction patterns. Vessel wall abnormalities and structural changes, such as endocardial/endothelial damage and dysfunction, were more frequently found in the LAA and subsequently increased the likelihood of thromboembolism [10]. The exact mechanism in which Xa-inhibitor-induced hemorrhagic pericardial effusion has not been identified. 

In our case, the patient was only on metoprolol for rate control before admission when he was found to have large hemorrhagic pericardial effusion, but was later placed on amiodarone for rhythm control. It is worth noting that amiodarone is mainly metabolized by cytochrome P450 (CYP)3A4 and is a potent inhibitor of CYP1A2, 2C9, 2D6, and 3A4 [11]. Although commonly prescribed together, drug-drug interactions between apixaban and amiodarone may lead to an increased risk of bleeding through CYP3A4 inhibition, which has also been seen in similar case studies [12].

Pericardial effusion is diagnosed by transthoracic echocardiogram (TTE). The degree of pericardial effusion relies on linear measurements of the largest width of the effusion at the end of the diastole. This system classifies effusion graded by size: small (<10 mm), moderate (10-20 mm), and large (>20 mm) [13]. As appreciated by the echocardiogram during the initial evaluation (Figure 3), the linear measurement of the effusion is 34.2 mm and is designated a large pericardial effusion.

In cases of large pericardial effusion, cardiac tamponade is the feared complication. As evidenced in this case, excessive fluid accumulation in the pericardium leads to compressive effects on the low-pressure right atrium, eventually resulting in restrictive filling of the cardiac chambers, which in turn leads to hypotension and cardiac arrest. With historical signs of sudden chest pain and associated pleurisy, suspicion of pericardial effusion is warranted. Physical examination findings of positional relief when leaning forward, Beck’s triad (hypotension, jugular venous distension, muffled heart sounds), and pulsus paradoxus may suggest cardiac tamponade. A cardiac electrocardiogram may show typical changes of PR-segment elevation in aVR or widespread T-wave inversions, followed by T-wave normalization. A bedside echocardiogram should be promptly performed to evaluate the extent of the effusion and diagnose cardiac tamponade [14]. 

The treatment of cardiac tamponade is pericardiocentesis. The procedure is performed to correct hypotension due to the decreased stroke volume from extrinsic compression of the heart chambers. In a hemodynamically unstable patient, there are no absolute contraindications for pericardiocentesis; however, in stable patients, relative contraindications exist, such as uncorrected coagulopathy, thrombocytopenia, and unknown chest anatomy [15]. Our case demonstrated a critically ill patient with hemodynamic instability requiring prompt intervention for life-saving care, which included activating the catheterization lab to perform pericardiocentesis under image guidance. 

Overall, not much data or subject population exists regarding DOACs, specifically apixaban, and their relationship to spontaneous hemorrhagic pericardial effusions, except for the increased risk of bleeding. Our case illustrates yet another example of this rare but observed phenomenon.

## Conclusions

Hemorrhagic pericardial effusions remain a rare yet consistently reported life-threatening complication with apixaban use. Although these effusions typically present with clinical syndromes that mimic heart failure or pneumonia, failure to recognize the condition early may lead to cardiac tamponade and death if not appropriately treated. Historical gathering, prompt physical examination, and ultrasound imaging are imperative to confirm the diagnosis, and emergent pericardiocentesis is required for cardiac tamponade. It is worth noting that upon initiating anticoagulative therapy with apixaban or other DOACs, thorough medication reconciliation is necessary to evaluate for any CYP3A4 inhibiting medications, which may instigate or predispose the patient to bleeding. Further clinical studies involving interval echocardiographic screening when implementing DOAC therapy to increase the likelihood of preventing deadly pericardial effusions should be investigated.
